# The Physiologist's Note-Book

**Published:** 1894-03

**Authors:** 


*The Physiologist's Note-Book.* By ALEX HILL, M.D. Pp. xii., 200. London: CHARLES GRIFFIN AND COMPANY, LIMITED. 1893.

Dr. Hill, though best known as an anatomist, has conducted for some years a tutorial class in Physiology at Cambridge, in a most successful way. Many an old Cambridge man looks back with gratitude to that class as of the very greatest aid to him, not only in the actual acquirement of facts, which was a small part of the matter, but because he there learned to understand the reasons and values of the facts, and how to systematise and classify his knowledge, to so store it away in his memory that it was always available for use when required.

Of course the value of a note-book lies in its compilation by the user of it himself, but we take it that by this book the author hopes to aid those whom he cannot personally reach. In the preface it is stated that the work is in no way meant to replace larger text-books or practical work in the laboratory, but to supplement these by helping the student to codify his knowledge. All such books have the drawback that they may be used simply as cram-books, and the acquirement of knowledge—if it can be called knowledge—in such a manner cannot be too strongly discouraged. Physiology, too, is the most difficult of all subjects to treat as it is treated in this Note-Book; it is often impossible to adequately discuss all the points about a disputed question in a brief manner under headings of *pro* and *con*. For instance, how difficult it is to give an intelligible account of Nutrition in brief abstract to a second or third year's student!

So far as this book is concerned, the work is well done—in fact, it could not be better done, if the difficulties of the subject are remembered, and that it is not intended for a treatise on Physiology. Taken as a whole, it is up to date in its information, and trustworthy in its statements. The diagrams are to be especially commended; they are clear, and deal in a very helpful way with just the points on which students need elucidation.

Many men have a really extensive knowledge of a subject, but no power of arranging their facts in an orderly manner; we believe a number of very good men fail in examinations simply from this cause, that they cannot get at their facts when they want them. To such men this book will be valuable, used in the way it is intended to be—as a supplement, and as a guide to the intelligent use of the larger text-books.

